# Internet Usage and Intrinsic Capacity in England

**DOI:** 10.1093/geroni/igaf122.2805

**Published:** 2025-12-31

**Authors:** Ethan Wang, Paola Zaninotto

**Affiliations:** University College London, London, England, United Kingdom; University College London, London, England, United Kingdom

## Abstract

The population aged 65 years and older in the United Kingdom is projected to grow from 11.8 million in 2016 to 20.4 million in 2066. However, digital disparities persist, with 46% of those aged 75 years and older in the United Kingdom not using the internet in 2020. Few studies have investigated the link between internet usage and intrinsic capacity, which is the combination of an individual’s physical and mental abilities. Data was from the 2012-2013 Wave 6 of the English Longitudinal Study of Aging (ELSA). The analysis sample included 4,143 participants aged 60 years and older. Internet usage was determined by respondents’ self-reported frequency in using the internet or email, categorized as high, medium, and low. Intrinsic capacity was modeled as a composite score of 14 variables: word recall, orientation in time, balance, chair rises, walking speed, upper mobility, lower mobility, eyesight, hearing, grip strength, body mass index, waist circumference, depressive symptoms, and life satisfaction. Linear regression was used to model associations of internet usage with intrinsic capacity. After full adjustment for demographics and health characteristics, participants with medium internet usage did not have an association with lower intrinsic capacity compared to the reference group (β:-0.07, 95%CI:-0.22, 0.07). Participants with low internet usage had an intrinsic capacity score 0.44 units lower compared to those with high internet usage (95%CI:-0.58, -0.29). Low internet usage is associated with lower intrinsic capacity among older adults in England. The risks of low technology usage to healthy aging should be investigated in future studies.

